# Examining the safe-haven and hedge capabilities of gold and cryptocurrencies: A GARCH and regression quantiles approach in geopolitical and market extremes

**DOI:** 10.1016/j.heliyon.2024.e40400

**Published:** 2024-11-15

**Authors:** Hanen Ben Ameur, Fouad Jamaani, Mohammed N. Abu Alfoul

**Affiliations:** a Department of Economics and Finance, College of Business Administration, Taif University, Saudi Arabia; b Department of Computing Technologies and Data Analytics, Ezymart Corporation Pty Ltd, Sydney, Australia

**Keywords:** Cryptocurrencies, Gold, Safe-haven, Hedge, COVID-19 pandemic geopolitical risks

## Abstract

This paper examines gold and cryptocurrencies' hedge and safe-haven capabilities against various downturns, including the COVID-19 pandemic and Geopolitical Risks (GPR), across different market conditions. The study covers a sample period from 2013 to 2021 at a daily frequency, employing the GARCH model and quantile regression with binary variables.

The empirical results indicate that neither gold nor cryptocurrencies can act as strong hedges against infectious disease pandemics. However, gold, Bitcoin, and Ethereum exhibit weak safe-haven abilities during geopolitical risks.

Using regression quantiles, the study finds that gold demonstrates a strong safe-haven against low and high Infectious Disease Epidemic Market Volatility (IDEMV) during extremely bearish and bullish markets. In contrast, Bitcoin and Ethereum act as strong safe havens only against low IDEMV during extreme bearish markets. Gold also shows a strong hedge propriety against extreme geopolitical events, while cryptocurrencies provide a weak hedge.

Overall, gold exhibits strong safe-haven properties against low and high Geopolitical tensions, while cryptocurrencies' hedging and safe-haven abilities vary across markets. These findings convey insights for investors and guidance to supervisors on the evolution of gold, Bitcoin, and Ethereum as safe-haven and hedge instruments during both bearish and bullish markets.

## 1 Introduction

During the COVID-19 pandemic, global financial systems were subjected to unprecedented strain [[1], [2], [3], [4]]. The rapid escalation of the pandemic led governments worldwide to implement stringent lockdown measures, restricting movement, enforcing city-wide shutdowns, and halting business activities. These actions significantly slowed economic growth on a global scale. Sharif et al. [5] characterized COVID-19 as the foremost global geopolitical shock of the 21st century. Caldara et al. [6] define geopolitical risks (GPR) as “risks associated with wars, tensions, terrorism, and international crises that affect international relations.” Such events introduce extreme risks to capital markets and profoundly influence investment portfolio decisions. During these turbulent periods, investors seek better safeguards for their asset portfolios using appropriate safe-haven tools [7]. Consequently, geopolitical risks are major factors influencing investment decisions [[8], [9], [10], [11]]. Extreme circumstances related to infectious diseases or geopolitical risks cause investors to become fearful, resulting in unexpected market movements that affect the volatility and returns of various financial securities. Ichev and Marinč [12] observed that the 2014–2016 Ebola outbreak significantly impacted investors' strategies. Al Mamun et al. (2020) distinguished between the impacts of the current COVID-19 pandemic and previous infectious diseases such as MERS in 2012, SARS in 2017, Ebola Virus Disease in 2014, and Zika in 2016 [13], highlighting that the COVID-19 pandemic crisis posed a far greater threat to capital markets as traditional hedging and safe-haven instruments behaved unusually [14]. Our study distinguishes itself from previous studies by focusing specifically on gold and cryptocurrencies and employing advanced statistical techniques such as quantile regression and GARCH models. These models are used to evaluate the performance of these assets under varying levels of market stress related to infectious diseases and geopolitical risks, offering a nuanced understanding of their performance in specific conditions. In contrast, other studies analyze a broader range of assets, including traditional currencies, across a wider timeframe and various global events. While our study offers refined insights into the conditions under which these assets act as hedges or safe havens, providing a focused analysis of the unique conditions under which gold and cryptocurrencies act as hedges or safe havens. This approach provides a refined and detailed perspective that complements the broader, event-driven approach that has been used in similar studies ([[15], [16], [17]], among others).

Our contribution is to examine the hedge and safe-haven properties of gold and two major cryptocurrencies—Bitcoin and Ethereum—against risks under various market conditions. Ethereum, as the second-largest cryptocurrency, along with Bitcoin, holds a dominant position in the cryptocurrency landscape, collectively making up nearly 70 % of the global market [18]. Specifically, we analyze the uncertainty associated with infectious diseases (IDEMV) and geopolitical risks (GPR) during several turbulent periods, including the COVID-19 crisis, Ebola Virus Disease in 2014, SARS in 2017, and Zika in 2016. Each asset's role may change depending on the type of market turbulence [19]. The study covers a sample from January 2, 2013, to November 30, 2021, on a daily frequency. We use a newly developed Infectious Disease Equity Market Volatility Tracker (IDEMV) while employing the GARCH model and quantile regression with binary variables. Our study employs two-dimensional criteria for hedges and safe havens: strong and weak investment assets: A weak (strong) hedge asset is an instrument that, on average, is not correlated (significantly positively correlated) with ID-EMV or GPR. A weak (strong) safe-haven status is defined as an uncorrelated instrument (significantly positively correlated) with ID-EMV or GPR on average during stressful periods.

The study results indicate that, compared to the IDEMV, neither gold nor cryptocurrencies can serve as strong hedges. However, Ethereum shows potential as a strong safe-haven asset. The GARCH model results indicate that Ethereum's safe haven coefficients are positive and statistically significant at the 10 % and 1 % levels. These findings suggest that Ethereum tends to act as a safe haven for IDEMV at the 90 % and 95 % quantiles, providing a strong safe haven against a pandemic crisis. In contrast, Bitcoin and gold show non-significant safe haven coefficients, indicating a weaker safe haven status during extreme market conditions. During periods of geopolitical risk, Gold, Bitcoin, and Ethereum demonstrate weak safe haven abilities. Furthermore, regression quantiles show that gold and Bitcoin can act as weak hedges during extreme bearish markets. Ethereum and Bitcoin exhibit strong safe-haven characteristics for lower IDEMV shocks in bearish markets, but this ability does not persist during extremely bullish markets. Only Gold remains a strong safe-haven asset against the highest IDEMV shocks.

In regard to against extreme geopolitical events, cryptocurrencies demonstrate weak hedging capabilities, especially during periods of low GPR. Their hedging properties are not consistent over time. Cryptocurrencies provide mixed results as both weak and strong safe-haven assets during extreme bearish and bullish markets.

In contrast, gold is a strong hedge and maintains strong safe-haven properties for lower and higher GPR shocks during extremely bearish or bullish markets. Those findings provide useful information to investors and financial advisors searching for how gold, bitcoin, and Ethereum evolved as hedge and safe-haven assets during the severe financial market panic caused by infectious diseases and geopolitical tensions. Finally, we use the newly developed Infectious Disease Equity Market Volatility Index proposed by Baker et al. [20] and the geopolitical risks (GPR) index that is newly developed by Caldara et al. [6] to investigate Gold and cryptocurrencies’ hedging and safe-haven properties against IDEMV and GPR, across various market conditions, employing GARCH approach and quantile regression with binary variables. This paper is organized as follows: Focusing on Bitcoin and Ethereum, Section 2 reveals the literature review on gold and cryptocurrencies as financial assets. Section 3 describes the data set, methodology, the GARCH approach, and the quantile regression model. Section 4 illustrates the major empirical results and their implications. The final section expresses the concluding remarks.

## 2 Literature review

### 2.1 The hedging and safe-haven characteristics of gold in turbulent periods

During uncertain political times or following an economic turmoil and financial event, gold's safe-haven potential occur more attention [[21], [22], [23]]. Research finds that other precious metals, such as silver, platinum, and palladium, are less valued since their safe-haven potential is only relevant in the short term [24]. However, Shahzad et al. [25] argue that gold's safe-haven properties may not hold consistently under different market conditions.

Nevertheless, research reports contrasted results regarding the safe-haven asset features of gold during the COVID-19 pandemic [21,[26], [27], [28]] Research finds that gold acts as a safe-haven asset and hedging tool for the G7 stock markets [26,[29], [30], [31], [32]]. Ali et al. [33] find that gold consistently exhibits safe-haven abilities in many advanced stock markets. Salisu et al. [32] show that gold acted as a safe-haven asset against oil price risks during the COVID-19 pandemic. Similarly, Triki and Maatoug (2021) demonstrate that gold serves as a good diversifier and safe-haven against the S&P 500, especially in the presence of high geopolitical tensions. In contrast, Będowska-Sójka and Kliber [34] and Cheema and Szulczuk [30] find that gold cannot always be a safe-haven asset during a pandemic.

### 2.2 The hedging and safe-haven characteristics of cryptocurrencies in the turbulent periods

Finance literature identifies cryptocurrencies such as Bitcoin as a prospective safe-haven asset [25,35]. Bitcoin is still the most widely known cryptocurrency, followed by Ethereum; both have the largest market capitalization. Bouri et al. [29] discover that gold is not a better shelter asset during the COVID-19 pandemic in distinction to bitcoin. In contrast, Kinateder et al. [7] reveal that most of the time, gold investments were more advantageous than bitcoin for stock markets during the current pandemic.

However, finance research argues that the COVID-19 pandemic positively impacts the cryptocurrency market's efficiency [26,36,37]. Some authors argue that Bitcoin can be considered a highly regarded instrument that is employed to surpass risks, including infectious disease crises and geopolitical risks [[38], [39], [40], [41]]). Research contends that Bitcoin was used as an asset to avoid GPR during great tension, such as the political tension in North Korea in 2017. Investors benefit from the Bitcoin market by reducing the risk of their portfolios [25,42,43]. On the occurrence of extreme geopolitical risk events, Su et al. [44] demonstrate that investors, to optimize their investments, can capitalize on the Bitcoin market. In contrast, other authors find that Bitcoin cannot be regarded as a safe-haven instrument due to its extreme volatility and riskiness, even in the long term [22,36,[45], [46], [47], [48]].

Table 1 presents a selected review of the role of gold and cryptocurrencies as hedge and safe-haven assets.

**Table 1:** Summary of previous empirical research.

Study	Sample period	Findings
**The hedging and safe-haven characteristics of Gold**		
Bouri et al. [29]	July 2010–February 2018	Gold is not a better choice compared to Bitcoin as a safe-haven asset during COVID-19.
Cheema and Szulczuk [30]	1990–2021	Showed that gold lost its safe-haven abilities during the pandemic.
Ji et al. [26]	August 2019–March 2020	Gold acted as a safe-haven asset for the MSCI equity index, the CoinDesk price index, and forex rates (USDCNY and EURUSD).
Akhtaruzzaman et al. [49]	December 2019–April 2020	During Phase I (December 31, 2019–March 16, 2020): gold acted as a safe-haven asset for stock markets during the pandemic. During Phase II (March 17–April 24, 2020): gold lost its safe-haven role.
Salisu et al. [32]	2016–2020	Gold played as a safe-haven asset against oil price during COVID-19.
Triki and Maatoug (2021)	1985–2018	Gold acts as a safe haven against the US stock market in the presence of geopolitical tensions.
Ali et al. [33]	2001–2018	In many advanced stock markets, gold appears to be the most reliable safe-haven asset.
Belguith et al [50]	November 2021–January 2023	Gold-backed cryptocurrencies possess both dynamic hedging and safe-haven abilities against the volatility of DeFi and NFT assets due to their stability grounded in the value of gold, making them a valuable tool for managing risks in the rapidly changing digital asset environment
Al-Nassar et al [51]	January 2016–September 2021	Gold remains a robust safe haven and hedge during crises such as the COVID-19 pandemic, outperforming Bitcoin.
**The hedging and safe-haven characteristics of cryptocurrencies**		
Aysan et al. [52]	July 2010–May 2018	Bitcoin can be considered a hedging tool against global geopolitical risks.
Kumar and Padakandla [53]	2015–2020	While Bitcoin provided mixed results, gold systematically displays safe-haven abilities for all the markets except the NSE in the long and short run.
Syuhada et al. [43]	September 2018–March 2021	Bitcoin's safe-haven functionality is inconsistent, especially during stormy periods like the COVID-19 crisis.
Corbet et al. [36]		During the COVID-19 outbreak, Bitcoin provide limited hedge or safe-haven advantages.
Conlon et al. [42]	March 2019–March 2020	Bitcoin failed to offer hedge or safe-haven abilities against the extreme bear market in the S&P 500 occasioned by the COVID-19 pandemic.
Shahzad et al. [25]	July 2010–February 2018	During extreme market conditions, Bitcoin and gold exhibit weak safe-haven abilities for stock market investments.
Bouri et al. [29]	August 2015–July 2018	Bitcoin, Ethereum, and Litecoin are hedges, particularly against Asian Pacific and Japanese equities.
Akhtaruzzaman et al. [54]	August 2011–November 2018	Bitcoin acts as an optimal hedge when diversified with stocks in the utility sector.
Charfeddine et al. [55]	July 2010–October 2018	Cryptocurrencies, such as Bitcoin, are often regarded as insufficient and weak hedging instruments.
Feng et al.,(2024)		Due to their stability and liquidity, cryptocurrencies can effectively serve as safe havens and hedges against global stock market fluctuations.
Hsu et al. [56]	August 2015–August 2022	Cryptocurrencies offer notable diversification benefits and can serve as hedges and safe havens under certain conditions, but their effectiveness is influenced by market structure and economic conditions.

### 2.3 Contributions to existing research

Our study is related to previous research, but it extends their work in several ways. Firstly, we investigate the role of gold and cryptocurrencies against various crises (not against assets such as stocks or energy). Much less attention has been paid to the ability of other cryptocurrencies to mitigate risks. The study by Będowska-Sójka and Kliber [34] is one of the exceptions.

Our paper extends previous studies by comparing the potential abilities of gold and cryptocurrencies against several crises, which are still scarce. To our knowledge, research on these particular topics is relatively recent and evolving. Thus, we posit the following research question: Do gold, bitcoin, or Ethereum possess hedge or safe-haven properties during highly tense periods associated with infectious diseases and geopolitical risks, respectively?

The sample covers nine years, from January 2013 to November 2021. Hence, it includes several crisis periods: Ebola Virus Disease in 2014, SARS in 2017, and Zika in 2016, as well as the most recent pandemic outbreak. Unlike most of the prior studies that consider the safe-haven abilities of Bitcoin only, we also examine other significant cryptocurrencies. The choice of Bitcoin and Ethereum cryptocurrencies is motivated by the fact that they have dominated the cryptocurrency market and have the highest capitalization [57]. We compare the hedge and safe-haven abilities of gold and two cryptocurrencies against IDEMV/GPR shocks during various market conditions and study whether the potential safe-haven or hedge coefficients are significantly changed if we take various bearish and bullish phases in the gold and cryptocurrency markets (for both smaller and larger IDEMV/GPR shocks). We apply the GARCH model and quantile regression using binary variables, which incorporate the average condition's extremes and distinct quantiles. (i.e., bearish, normal, and bullish markets). In doing so, we evaluate the weak and strong hedge or safe-haven properties of gold and cryptocurrencies for both lower and bigger IDEMV/GPR shocks.

## 3 Methodology, data, and empirical analysis

### 3.1 Estimations via GARCH model with binary variables

We use both quantile regression and a generalized autoregressive conditional heteroskedasticity (GARCH) framework with binary variables based on the study of Baur and Lucey [21] and Bouri et al. [35] to examine and compare the hedge and safe-haven abilities of gold and two major cryptocurrencies—Bitcoin and Ethereum––against risks across various market conditions related to the uncertainty associated with infectious diseases (IDEMV) and geopolitical risks (GPR). Those two methods are chosen because of their specific strengths in handling financial and economic data characteristics. GARCH models excel in capturing volatility clustering, heteroskedasticity, and leverage effects, making them ideal for forecasting volatility and understanding financial market dynamics related to IDEMV and GPR. Quantile regression, on the other hand, provides a comprehensive view by estimating the conditional median or other quantiles, offering robustness to outliers and the ability to analyze heterogeneous effects across the distribution. These methodologies provide unique insights and robustness that are particularly suited to the intricate nature of financial and economic data, making them preferred choices over other methodologies. Using the GARCH framework as calculated in Equations (1), (2) allows us to estimate the magnitude of the change in correlation and introduce a significant error-correction term after COVID-19. The maximum likelihood technique is used to estimate the following models:

(1) $$r_{t=}\alpha +\lambda r_{t-1}+\beta_{0}r_{i,t}+\beta_{1}\;D\left(r_{i,q90}\right)r_{i,t}+\beta_{2}\;D\left(r_{i,q95}\right)r_{i,t}+\beta_{3}\;D\left(r_{i,q99}\right)r_{i,t}+\varepsilon_{t}$$

(2) $$\sigma_{t}^{2}=\theta_{0}+\theta_{1}\varepsilon_{t-1}^{2}+\theta_{2}\sigma_{t-1}^{2}$$

In Equation (1), r_t_ denotes the return of gold, Bitcoin, or Ethereum, $r_{i,t}$ reflects the change in the IDEMV or GPR index. $D\left(r_{i,q90}\right)$ is the binary variable of the 90 % quantile, which implies that when the change of IDEMV or GPR is less than the 90 % quantile, the binary variable takes 0; elsewhere, it takes 1. The building methods are similar to $D\left(r_{i,q95}\right)$ and $D\left(r_{i,q99}\right)$ which indicate the 95 % and 99 % binary variables, as mentioned in eq. (1) correspondingly.

Our paper investigates if gold, Bitcoin, or Ethereum may serve as a hedge or safe-haven against IDEMV or GPR events under average conditions., The hedge or safe-haven in this study should have the following features: When the IDEMV or GPR index increases, the asset's value is still on the rise (not falling).

So, this study explores the characteristics of financial instruments that protect against market downturns (hedges) or provide stability during such periods (safe-havens). A weak (strong) hedge is an instrument with low/high average correlations to market volatility (IDEMV or GPR); a weak (strong) safe haven is an instrument with low/high average correlations to market volatility during stressful times. It refines the criteria established by Iqbal and Finance [58] by examining the correlations between instruments and market volatility under various stress levels and identifying if $\beta_{0}$ > 0, holds, gold, Bitcoin, or Ethereum is deemed a hedge against market volatility; if $\sum_{i=0}^{1}\;\beta_{i}>0$, gold, Bitcoin or Ethereum is a Safe-haven against IDEMV or GPR at the 90th percentile of market stress. ; if $\sum_{i=0}^{2}\;\beta_{i}>0$, gold, Bitcoin or Ethereum is a safe-haven at the 95th percentile; if $\sum_{i=0}^{3}\;\beta_{i}>0$, gold, Bitcoin or Ethereum is a safe-haven at 99th percentiles.

In fact, different stress levels are characterized by specific quantiles (e.g., 90th, 95th, 99th percentiles), indicating the severity of market downturns.

### 3.2 Estimations via quantile regression with binary variables

Where Y represents the return of gold, Bitcoin or Ethereum, and X indicate the change of the IDEMV or GPR index. Hence, we take Y in Equation (3) as a random variable with a real-valued cumulative distribution function F_Y_(y) = P(Y ≤ y), the ξ*th* conditional quantile of Y given X = x is defined as:

(3) $$Q_{Y|x}\left(\xi \right)=F_{Y|x}^{-1}\;\left(\xi \right)=\inf\;\left\{y:F_{y|x}\left(y\right)\geq \xi \;\right\},\xi \in \left[0,1\right]$$

Where $Q_{Y|x}\left(\xi \right)=x^{\prime }\beta_{\left(\xi \right)},\beta_{\left(\xi \right)}$ is the coefficient vector for x at the ξth quantile, thus

(4) $${\hat{\beta }}_{\left(\xi \right)}=\mathrm{argmin}\sum \rho_{\left(\xi \right)}\;\left(y_{i}-x^{\prime }\beta \right)$$

$\rho_{\xi }\left(y\right)=y\left(\xi -I\left(y<0\right)\right),I\left(.\right)$ is the indicator function. On this basis, we set up the following model to test whether gold, Bitcoin or Ethereum is the hedge or safe-haven for IDEMV or GPR at different quantiles:

(5) $$Q_{Y|x}\left(\xi \right)=\mu +\beta_{0\left(\xi \right)}r_{i,t}+\beta_{1\left(\xi \right)}D\left(r_{i,q90}\right)r_{i,t}+\beta_{2\left(\xi \right)}D\left(r_{i,q95}\right)r_{i,t}+\beta_{3\left(\xi \right)}D\left(r_{i,q99}\right)r_{i,t}+e_{t}$$

For $D\left(r_{i,q90}\right)$, $D\left(r_{i,q95}\right)$, and $D\left(r_{i,q99}\right)$ as mentioned in Equation (5) denote binary variables with values 1 if the IDEMV or GPR index change surpass the corresponding quantiles and 0 otherwise. The safe-haven and hedge capabilities of gold, Bitcoin, or Ethereum are calculated in the same manner under the average condition at ξth quantile.

### 3.3 Data

We used the prices of gold and Bitcoin from January 2, 2013, to November 30, 2021. The frequency of the data is daily. Ethereum prices are used from March 10, 2016, to November 30, 2021. We convert prices to returns by taking their logarithmic differences^1^ Δ ln(X_t_)= (ln(X_t_)- ln(X_t−1_))∗100. Our analysis focuses on two leading and decentralized digital currencies in the global market: Bitcoin and Ethereum [34]. The beginning date of Ethereum is based on the availability of data on Ethereum prices. The gold, Bitcoin, and Ethereum price data are obtained from the Investing Website. All variables are studied in US dollar terms.

The year 2021 corresponds to the conclusion of major economic disruptions caused by the COVID-19 pandemic. Including data up to 2021 allows us to capture the full impact of the pandemic while maintaining a consistent analysis period.

In this study, we introduce the newly constructed infectious disease equity market volatility (IDEMV) index of Baker et al. [1]. The index is published at a daily frequency and included as an exogenous factor and involves several factors, such as political tensions and friction.

We also consider the recent GPR index of adverse geopolitical events and associated risks at a daily frequency, constructed by Caldara et al. [6], based on ten newspapers. Caldara et al. [6] define geopolitical risk as the “risk associated with wars, tensions, terrorism, and international crises that affect international relations.” In such cases, elevated levels of risk, instability, and uncertainty are generated, affecting economic conditions, global market agents, and sentiment [59,60]. These indexes allow for examining the effects of various diseases and geopolitical tensions on the gold and cryptocurrency markets. The summary statistics for the daily returns of Gold, Bitcoin and Ethereum, and the changes of IDEMV and GPR indexes are presented in Table 2.

**Table 2:** Summary statistics.

Variable	IDEMV	GPR	GOLD	BITCOIN	Ethereum
Mean	0.003619	0.002706	0.002181	0.256866	0.285807
Median	0.000000	−0.087872	0.007553	0.199088	0.134418
Maximum	37.44000	236.1476	11.96911	147.4180	25.85994
Minimum	−34.7300	−240.0317	−11.79703	−84.88287	−58.96385
Std. Dev.	4.321428	45.25236	1.619857	5.757552	5.750571
Skewness	0.159781	0.099979	−0.000898	4.432602	−0.616285
Kurtosis	21.15410	5.198404	18.43144	168.3621	11.35635
Jarque-Bera	32225.57∗∗∗	476.3321∗∗∗	23277.14∗∗∗	3719274.∗∗∗	6216.181∗∗∗
Probability	0.000000	0.000000	0.000000	0.000000	0.000000
KPSS	0.044119∗∗∗	0.131427∗∗∗	0.267689∗∗∗	0.156976∗∗∗	0.166213∗∗∗
ADF	−17.521∗∗∗	−23.54386∗∗∗	−31.82113∗∗∗	−21.68356∗∗∗	−48.09476∗∗∗
P-P	−262.523∗∗∗	−689.3853∗∗∗	−66.73165∗∗∗	−56.78300∗∗∗	−48.04086∗∗∗

Notes: ∗, ∗∗ and ∗∗∗ indicate significance at 10 %, 5 %, and 1 % levels, respectively.

Our descriptive analysis shows that gold has the lowest positive mean return and GPR has the highest standard deviation; all variables have a positive mean return. The standard deviation fluctuates between 1.619 and 42.260 for the gold returns and the geopolitical risk index figures, respectively. This is because of the safe-haven nature of gold, which shows the slightest volatility. Cryptocurrencies and the indices of uncertainty have excess kurtosis and non-zero skewness. The Jarque–Bera test indicates that all series are non-normally distributed. The results of the Kwiatkowski-Phillips-Schmidt-Shin (KPSS), Augmented Dickey-Fuller (ADF), and Phillips-Perron tests for thorough stationarity analysis revealed that the selected variables of the study are stationary at the first difference [61]. This suggests the appropriateness of applying GARCH and a quantile-based approach.

## 4 Results

### 4.1 The effects of infectious diseases on gold, bitcoin, and Ethereum returns via the GARCH model with binary variables

Table 3 shows the estimates results via the GARCH model and the resulting standard errors, to examine the hedge and safe-haven roles of gold, Bitcoin, and Ethereum against the change of IDEMV at the average condition. Relying on the GARCH model, we observe that for Gold: $\beta_{0}$ < 0 and significant at the 10 % level indicate that gold cannot serve as a strong hedge against IDEMV; $\sum_{i=0}^{1}\beta_{i}$ >0 suggests that gold is a weak safe-haven for IDEMV at the 90 % quantile; in other cases, gold is not a safe-haven. Regarding Bitcoin: $\beta_{0}$ < 0 suggests that Bitcoin is not a hedge against IDEMV; $\sum_{i=0}^{k}\beta_{i}$ >0 (k = 1, 2) indicates that Bitcoin is a weak safe-haven for IDEMV at 90 % and 95 % quantiles.

**Table 3:** GARCH model with binary variables results for the effects of the change of IDEMV index on gold, Bitcoin, and Ethereum returns.

Parameters	λ	$\beta_{0}$	$\sum_{i=0}^{1}\beta_{i}$	$\sum_{i=0}^{2}\beta_{i}$	$\sum_{i=0}^{3}\beta_{i}$	$\theta_{0}$	$\theta_{1}$	$\theta_{2}$
*Panel A: Gold*								
	−0.135∗∗∗ (0.0301)	−0.013217∗ (0.007422)	0.0319 (0.0347)	−0.01806 (0.0165)	−0.0047 (0.00937)	0.806∗∗∗ (0.158)	0.195∗∗∗ (0.037)	0.478∗∗∗ (0.066)
*Panel B: Bitcoin*								
	−0.0202 (0.0203)	−0.015 (0.0137)	0.1019 (0.059)	0.099 (0.0108)	−0.029298 (0.0245)	0.709∗∗∗ (0.03203)	0.1808∗∗∗ (0.006697)	0.8088∗∗∗ (0.004962)
*Panel B: Ethereum*								
	−0.0356 (0.0294)	−0.0505∗∗ (0.0221)	0.196∗ (0.142)	0.0702∗∗∗ (0.038)	−0.004 (0.0288)	2.351∗∗∗ (0.512)	0.1299∗∗∗ (0.0183)	0.803∗∗∗ (0.027)

Notes: ∗∗∗, ∗∗ and ∗ denote significance at 1 %, 5 %, and 10 % levels, respectively. The estimates are obtained after estimating the GARCH model.

Similarly, for Ethereum: $\beta_{0}$ < 0 suggests that Ethereum does not act as a hedge against IDEMV; however, $\sum_{i=0}^{k}\beta_{i}$ >0 (k = 1, 2), which is significant at the 10 % and 1 % levels, indicates that Ethereum is a strong safe-haven for IDEMV at the 90 % and 95 % quantile. To summarize, neither cryptocurrencies nor gold can exhibit as a strong hedge against IDEMV; though Ethereum can serve as a strong safe-haven, gold and Bitcoin act as weak safe-havens against IDEMV.

### 4.2 The effects of infectious diseases on gold, bitcoin, and Ethereum returns were examined via quantile regression with binary variables

We estimate seven quantiles, from the lowest (0.1) to the highest (0.95), in order to divide the market into three types, i.e., bearish, normal, and bullish. Table 4 shows how the various influences of infectious diseases on gold, Bitcoin, and Ethereum returns differ at lower and higher quantiles. The empirical results below indicate that gold can act as a weak hedge against the lower IDEMV condition, with a hedge coefficient $\beta_{0}$ > 0 at the lower and medium quantiles (10 % and 40 %), while it cannot serve as a hedge asset in other cases. At the lower $\sum_{i=0}^{1}\beta_{i}$ > 0 at the 5 % significant level on the 10 % quantile, this indicates that gold is a strong safe-haven for lower IDEMV shocks, while the coefficient turns out to be negative and significant at 5 % and 1 % on the 10 % quantile. At the higher quantiles (70 %, 85 %, and 95 %), the most safe-haven coefficients are positive, and at the 5 % significant level on the 95 % quantile, they indicate that gold is a strong safe-haven for upper IDEMV shocks. These results suggest that IDEMV shocks lead to increased gold returns in both bearish and bullish markets.

**Table 4:** Quantile regression with binary variables results for the effects of the change of IDEMV index on gold returns.

Quantile	10 %	25 %	40 %	55 %	70 %	85 %	95 %
*Panel A: Gold*							
$\beta_{0}$	0.000484 (0.00706)	−0.0014 (0.0049)	0.002 (0.005)	−0.0034 (0.0089)	−0.0086 (0.007)	−0.028 (0.026)	−0.018 (0.0312)
$\sum_{i=0}^{1}\beta_{i}$	0.074∗∗ (0.032)	0.035 (0.025)	0.007 (0.018)	0.0026 (0.0255)	0.0388 (0.041)	0.042 (0.038)	−0.041 (0.092)
$\sum_{i=0}^{2}\beta_{i}$	−0.0902∗∗ (0.038)	−0.0295∗ (0.022)	−0.0216∗ (0.012)	−0.011 (0.0109)	0.015 (0.017)	0.0055 (0.015)	0.096∗∗ (0.046)
$\sum_{i=0}^{3}\beta_{i}$	−0.0488∗∗∗ (0.0116)	−0.027 (0.017)	−0.0125 (0.0117)	−0.0032 (0.011)	0.0068 (0.01004)	−0.007209 (0.0065)	0.0304 (0.0072)

Notes: ∗∗∗, ∗∗ and ∗ denote significance at 1 %, 5 %, and 10 % levels, respectively.

Table 5 provides the quantile regression model to explore the effects of the change in the IDEMV index on bitcoin returns. The hedge coefficient $\beta_{0}$ > 0 at the lower and medium quantiles (10 %, 25 %, and 40 %) suggests that bitcoin can act as a weak hedge against the lower IDEMV situation. In contrast, it could not be evolved as a hedging asset otherwise. At the lower quantiles (10 % and 25 %), $\sum_{i=0}^{2}\beta_{i}$ >0, the coefficients are positive and statistically significant at 1 % and 5 %, respectively, which indicates that bitcoin can act as a strong safe-haven during its extreme bearish market for lower IDEMV shocks. While the safe-haven coefficients $\sum_{i=0}^{k}\beta_{i}$ <0 (k = 1, 2, 3) are negative at the highest quantiles (85 % and 95 %), which suggests that Bitcoin does not offer safe-haven abilities during its extremely bullish markets for bigger IDEMV shocks.

**Table 5:** Quantile regression with binary variables results for the effects of the change of IDEMV index on Bitcoin returns.

Quantile	10 %	25 %	40 %	55 %	70 %	85 %	95 %
*Panel A: Bitcoin*							
$\beta_{0}$	0.032 (0.021)	0.031 (0.025)	0.00729 (0.0165)	−0.02275 (0.0201)	−0.0805∗∗∗ (0.017)	−0.0808∗∗ (0.032)	−0.024 (0.062)
$\sum_{i=0}^{1}\beta_{i}$	0.1602 (0.2309)	0.059 (0.069)	0.023 (0.054)	−0.0023 (0.055)	0.062 (0.083)	0.1112 (0.151)	0.016 (0.292)
$\sum_{i=0}^{2}\beta_{i}$	0.131∗∗∗ (0.032)	0.031∗ (0.016)	−0.013 (0.017)	0.0013 (0.0229)	0.0105 (0.03)	−0.027 (0.054)	−0.079 (0.106)
$\sum_{i=0}^{3}\beta_{i}$	−0.037603 (0.0223)	−0.022 (0.029)	−0.021∗ (0.011	−0.219 (0.0171)	−0.023 (0.0222)	−0.042 (0.0404)	−0.052 (0.078)

Notes: ∗∗∗, ∗∗ and ∗ denote significance at 1 %, 5 %, and 10 % levels, respectively.

Table 6 reports, via quantile regression with binary variables, the effects of the change of the IDEMV index on the return of Ethereum. $\beta_{0}$ < 0, in all the cases, suggests that Ethereum cannot act as a hedge against the IDEMV condition. At the lower, medium and higher quantiles (40 %, 55 %, and 70 %) $\sum_{i=0}^{1}\beta_{i}$ >0 and significant at the 1 % level indicate that Ethereum acts as a strong Safe-haven against IDEMV chocks; the same is true for the coefficients at the medium and higher quantiles, while the safe-haven coefficients turn to be negative $\sum_{i=0}^{k}\beta_{i}$ < 0 (k = 1, 2, 3) and significant at the highest quantiles (85 % and 95 %), this suggests that Ethereum could not serve as a strong safe-haven during its extremely bullish market for bigger IDEMV shocks. The safe-haven coefficients are positive at the lower quantiles (10 % and 25 %) and significant in some cases, indicating that Ethereum is a strong safe-haven for lower IDEMV shock. The safe-haven coefficients reveal that in numerous cases, Ethereum, gold and Bitcoin can be used only as a strong safe-haven against IDEMV during the bearish market. However*,* Ethereum could not serve as a safe-haven during its extremely bullish market for bigger IDEMV shocks. Specifically, the effects of IDEMV on cryptocurrencies are significantly positive at the lower quantiles and significantly positive at the upper quantiles for the gold returns. Bitcoin and Ethereum demonstrate only safe-haven capabilities, particularly in response to low IDEMV during extreme bearish markets. This partially aligns with Corbet et al. [36], who found limited safe-haven properties in Bitcoin, especially under certain market conditions. However, our findings contrast with claims from studies like Shahzad et al. [25] and Syuhada et al. [43] that cryptocurrencies can act as safe-havens under specific circumstances.

**Table 6:** Quantile regression with binary variables results for the effects of the change of IDEMV index on Ethereum returns.

Quantile	10 %	25 %	40 %	55 %	70 %	85 %	95 %
*Panel A: Ethereum*							
$\beta_{0}$	−0.021 (0.0428)	−0.013 (0.0209)	−0.0037 (0.019)	−0.055∗ (0.033)	−0.057∗∗∗ (0.021)	−0.046 (0.031)	−0.036 (0.084)
$\sum_{i=0}^{1}\beta_{i}$	0.148 (0.259)	0.143 (0.129)	0.293∗∗∗ (0.107)	0.360∗∗∗ (0.1365)	0.366∗∗∗ (0.118)	0.229 (0.186)	0.042 (0.54)
$\sum_{i=0}^{2}\beta_{i}$	0.113 (0.065)	0.096∗∗∗ (0.028)	0.056∗∗ (0.023)	0.036 (0.026)	0.09 (0.049)	−0.017 (0.042)	−0.193∗∗∗ (0.065)
$\sum_{i=0}^{3}\beta_{i}$	0.02207 (0.021)	−0.044∗∗ (0.0207)	−0.026 (0.023)	−0.036 (0.025)	−0.026 (0.027)	−0.055∗∗∗ (0.014)	−0.127∗∗∗ (0.016)

Notes: ∗∗∗, ∗∗ and ∗ denote significance at 1 %, 5 %, and 10 % levels, respectively.

For instance, while some papers (e.g., Ref. [35]) suggest potential safe-haven capabilities in Bitcoin, our analysis, along with others (e.g., Wang et al., 2019, [48]), shows that cryptocurrencies still lack reliability as consistent safe-haven assets, particularly in response to major market crises like COVID-19.

### 4.3 The effects of geopolitical risks on gold, bitcoin, and Ethereum returns examined via the GARCH model with binary variables

Table 7 presents the effects of geopolitical risks on gold, Bitcoin, and Ethereum returns via the GARCH model. The results show that the hedge coefficient for Gold: $\beta_{0}$ > 0 and significant at the 1 % level, indicating that gold acts as a strong hedge against geopolitical tenses GPR; $\sum_{i=0}^{k}\beta_{i}$ >0 (k = 1, 2, 3). Suggests that gold is a weak safe-haven for GPR at the 90 %, 95 %, and 99 % quantiles. For Bitcoin $\beta_{0}$ > 0 indicates that bitcoin acts as a weak hedge against geopolitical tenses. Similarly, the safe-haven coefficient $\sum_{i=0}^{k}\beta_{i}$ >0 (k = 1, 2, 3) indicates that bitcoin is a weak safe-haven at GPR at the 95 % and 99 % quantiles. In contrast, the hedge coefficient for Ethereum $\beta_{0}$ < 0 indicates that Ethereum does not act as a hedge against GPR. At quantile 95 % $\sum_{i=0}^{2}\beta_{i}$ < 0 and significant at the 10 % level, this suggests that Ethereum is not a safe-haven against GPR; in other cases, $\sum_{i=0}^{3}\beta_{i}$ >0 suggests that Ethereum acts as a weak safe-haven for GPR at 90 % and 99 % quantiles. The results suggest that only gold can serve as a strong hedge against GPR; Bitcoin is a weak hedge, and Ethereum is not a hedge against geopolitical tenses. However, gold, Bitcoin, and Ethereum can serve as weak safe-havens during geopolitical risks.

**Table 7:** GARCH model with binary variables results for the effects of the change of GPR index on gold, Bitcoin, and Ethereum returns.

Parameters	λ	$\beta_{0}$	$\sum_{i=0}^{1}\beta_{i}$	$\sum_{i=0}^{2}\beta_{i}$	$\sum_{i=0}^{3}\beta_{i}$	$\theta_{0}$	$\theta_{1}$	$\theta_{2}$
*Panel A: Gold*								
	−0.1299∗∗∗ (0.0307)	0.0026∗∗∗ (0.000751)	0.003019 (0.002473)	0.00177 (0.002263)	0.001182 (0.002482)	0.785∗∗∗ (0.03573)	0.1888∗∗∗ (0.0157)	0.490044∗∗∗ (0.0216)
*Panel B: Bitcoin*								
	−0.0258 (0.019)	0.000854 (0.0013)	−0.0014 (0.0044)	0.00228 (0.0027)	0.002287 (0.003807)	0.7056∗∗∗ (0.0328)	0.18007∗∗∗ (0.0064)	0.8107∗∗∗ (0.00501)
*Panel B: Ethereum*								
	−0.039 (0.025)	−0.003533 (0.002)	0.00296 (0.0058)	−0.007134∗ (0.00662)	0.00559 (0.0052)	2.2426∗∗∗ (0.2744)	0.1304∗∗∗ (0.0095)	0.8077∗∗∗ (0.0148)

Notes: ∗∗∗, ∗∗ and ∗ denote significance at 1 %, 5 %, and 10 % levels, respectively.

### 4.4 The effects of geopolitical risks on gold, bitcoin, and Ethereum returns examined via quantile regression with binary variables

Table 8 displays the results of the quantile regression model examining the relationship between the return of gold and the change of the GPR index. The analysis reveals various influences of geopolitical risks on gold, bitcoin, and Ethereum returns. $\beta_{0}$ > 0 at the lower and medium quantiles (10 %, 25 %, 40 %, and 55 %) suggest that gold is a weak hedge against lower GPR chocks, while the hedge coefficient at the 1 % significant level on the 95 % quantile indicates that gold can play as a strong hedge for larger GPR chocks. These results are in line with the results of the study by Triki and Maatoug (2021).

**Table 8:** Quantile regression with binary variables results for the effects of the change of GPR index on gold returns.

Quantile	10 %	25 %	40 %	55 %	70 %	85 %	95 %
*Panel A: Gold*							
$\beta_{0}$	0.0048 (0.002)	0.00179 (0.000685)	0.00074 (0.00055)	0.000255 (0.000518)	−0.000441 (0.000592)	0.000475 (0.000829)	0.0043∗∗∗ (0.0015)
$\sum_{i=0}^{1}\beta_{i}$	0.00602∗ (0.0033)	0.0026∗ (0.001359)	0.001144 (0.001337)	0.00222 (0.001604)	0.0036∗∗ (0.0018)	0.00015 (0.0001888)	0.0228∗∗ (0.011)
$\sum_{i=0}^{2}\beta_{i}$	0.0016 (0.0028)	0.000858 (0.00094)	−0.00024 (0.000932)	0.000729 (0.00109)	−0.00046 (0.00111)	0.000416 (0.00222)	0.029∗∗∗ (0.005177)
$\sum_{i=0}^{3}\beta_{i}$	−0.0007 (0.00204)	0.000406 (0.00098)	0.000638 (0.000916)	0.0001 (0.000866)	−0.000189 (0.0001205)	0.0011 (0.002485)	0.003782 (0.004596)

Notes: ∗∗∗, ∗∗ and ∗ represent significance at 1 %, 5 %, and 10 % levels, respectively.

$\sum_{i=0}^{1}\beta_{i}$ >0 and significant at the 10 % significant level on the 10 % and 25 % quantiles, which suggests that gold is a strong safe-haven at lower GPR chocks. The safe-haven coefficients $\sum_{i=0}^{k}\beta_{i}$ > 0 (k = 1, 2, 3) at the 95 % quantile and significant at 5 % and 1 %, demonstrating that gold can function as a strong safe-haven for the higher GPR chocks. Similarly, the gold market is bullish, and gold increases its returns during more uncertain times. An increase in the level of IDEMV has also had a positive impact on gold returns. Gold returns increase, conveying characteristics of a safe-haven during periods of crisis. This finding is in line with those of Wu et al. [62] and Triki and Maatoug (2021), who find that in the face of high political tensions, investors strive to hedge against excessive risks by diversifying more, frequently by increasing the proportion of gold (a safe-haven instrument) in their portfolios. Our findings reveal the strong safe-haven role of gold during times of increased geopolitical risk and market stress, which is consistent with previous research by Triki and Maatoug (2021),Salisu et al. [32], Al-Nassar et al. [51] and Foroutan, P., & Lahmiri, S. [28], who established gold's consistent safe haven capabilities throughout Market turmoil. Similar to previous research, we discovered that gold provides powerful protection against severe market situations, particularly during bearish and bullish times with large geopolitical risk (GPR) shocks.

According to the results in Table 9, Table 10, we provide the impacts of the change of the GPR index on the return of Bitcoin, and the return of Ethereum, via the quantile regression model, respectively.

**Table 9:** Quantile regression with binary variables results for the effects of the change of GPR index on Bitcoin returns.

Quantile	10 %	25 %	40 %	55 %	70 %	85 %	95 %
*Panel A: Bitcoin*							
$\beta_{0}$	−0.0047 (0.0053)	−0.0025 (0.001846)	−0.0015 (0.0013)	−0.00041 (0.0013)	−0.00062 (0.0022)	−0.0012 (0.0037)	0.000373 (0.00907)
$\sum_{i=0}^{1}\beta_{i}$	0.0067 (0.018)	0.00502 (0.003)	−0.0014 (0.0033)	0.0027 (0.00346)	0.000108 (0.0056)	0.0110 (0.0094)	0.0066 (0.0227)
$\sum_{i=0}^{2}\beta_{i}$	0.000156 (0.0083)	−0.0046 (0.0038)	−0.0025 (0.002)	0.00042 (0.0027)	0.00182 (0.0044)	0.0076 (0.007)	0.032937∗ (0.017)
$\sum_{i=0}^{3}\beta_{i}$	0.0078∗∗∗ (0.002827)	0.0059 (0.0048)	0.0047 (0.0031)	0.0037 (0.0032)	0.0052 (0.0052)	0.0101 (0.008856)	0.000102 (0.021)

Notes: ∗∗∗, ∗∗ and ∗ represent significance at 1 %, 5 %, and 10 % levels, respectively.

**Table 10:** Quantile regression with binary variables results for the effects of the change of GPR index on Ethereum returns.

Quantile	10 %	25 %	40 %	55 %	70 %	85 %	95 %
*Panel A: Ethereum*							
$\beta_{0}$	−0.012∗∗ (0.006)	−0.007∗∗ (0.003)	−0.0031 (0.0028)	−0.0033 (0.0029)	−0.0031 (0.0039)	0.006086 (0.0061)	0.00767 (0.0097)
$\sum_{i=0}^{1}\beta_{i}$	0.016 (0.022)	0.0084 (0.008405)	0.0025 (0.0053)	−0.000558 (0.0056)	−0.002065 (0.0106)	−0.00095 (0.0105)	−0.000873 (0.028)
$\sum_{i=0}^{2}\beta_{i}$	0.0105 (0.009)	0.0026 (0.0048)	−0.000975 (0.0049)	−0.000601 (0.007)	0.0134 (0.0085)	0.0008491 (0.008591)	0.0385∗∗ (0.016)
$\sum_{i=0}^{3}\beta_{i}$	0.0035 (0.027)	0.0043 (0.0078)	0.00416 (0.0041)	−0.000004 (0.0045)	−0.000451 (0.0125)	0.0237 (0.017)	0.0074 (0.0134)

Notes: ∗∗∗, ∗∗ and ∗ represent significance at 1 %, 5 %, and 10 % levels, respectively.

For Bitcoin, $\beta_{0}$ <0 at the lower and medium quantiles (10 %, 25 %, 40 %, and 55 %) suggests that bitcoin cannot act as a hedge against the lower GPR condition; however, at the 95 % quantile, the coefficient turned out to be positive $\beta_{0}$ > 0 suggests that bitcoin can be a weak hedge for the higher GPR condition. The safe-haven coefficients $\sum_{i=0}^{k}\beta_{i}$ >0 (k = 1, 2, 3) at a 1 % significant level on the 10 % quantile suggests that bitcoin is a strong safe-haven at lower GPR conditions. While at the medium and higher quantiles (25 %, 40 %, 55 %, 70 %, 95 %), it turned out to be a weak safe-haven against geopolitical chocks. At the 95 % quantile, $\sum_{i=0}^{2}\beta_{i}$ >0 at the 10 % significant level on the 95 % quantile suggests that bitcoin is a strong safe-haven at a higher GPR condition.

Regarding Ethereum in Table 10, the hedge coefficient $\beta_{0}$ < 0 at the lower and medium quantiles (10 %, 25 %, 40 %, and 55 %) suggests that Ethereum cannot act as a hedge against the lower GPR condition; however, at higher GPR conditions, $\beta_{0}$ > 0 indicates that Ethereum can serve as a weak hedge for GPR at 85 % and 95 % quantiles. The safe-haven coefficients are positive at the lower quantiles, which suggests that Ethereum could act as a weak safe-haven during its extreme bearish markets for the lower GPR shocks. $\sum_{i=0}^{2}\beta_{i}$ >0 at a 5 % significant level on the 95 % quantile, which indicates that Ethereum is a strong safe haven at higher GPR conditions. The significant positive effects of GPR on cryptocurrencies and gold illustrate that each can be considered an instrument to mitigate risks linked to severe geopolitical events.

**Fig. 1:**
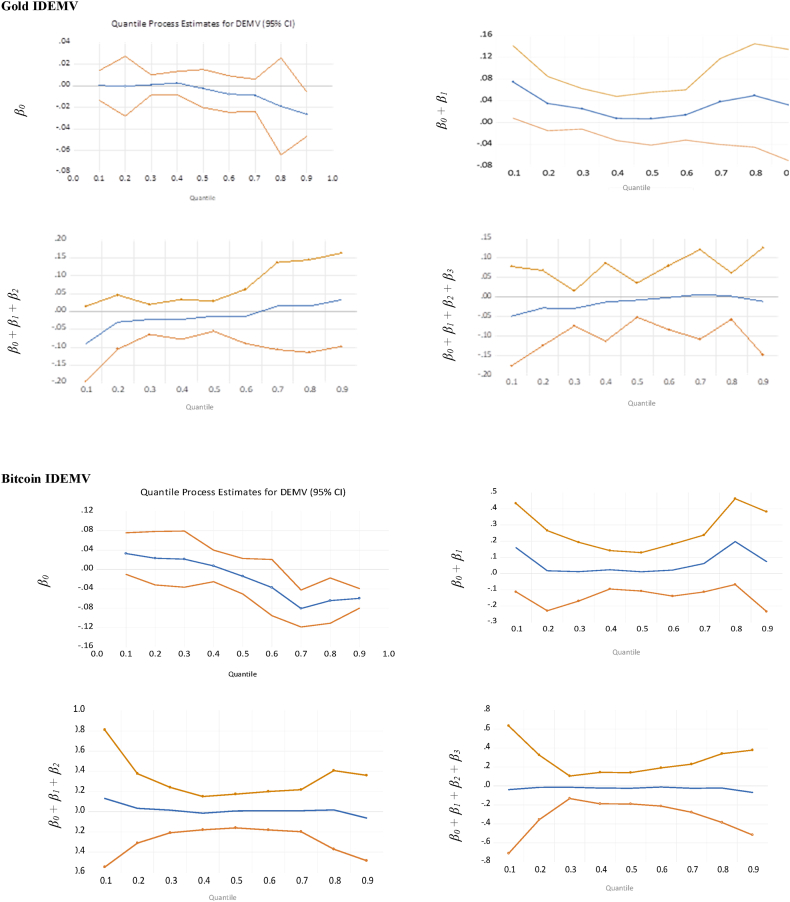

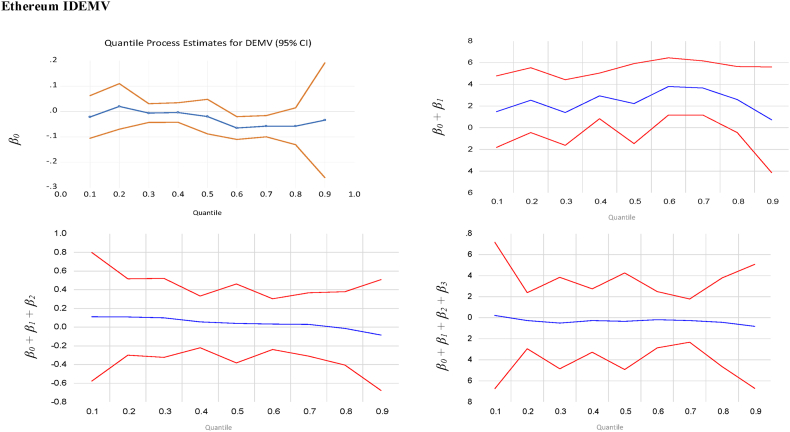
Quantile regression results with 95 % confidence bands for the effects of the change of IDEMV on Gold, Bitcoin and Ethereum at extreme 90 %, 95 %, and 99 % quantiles of IDEMV.

**Fig. 2:**
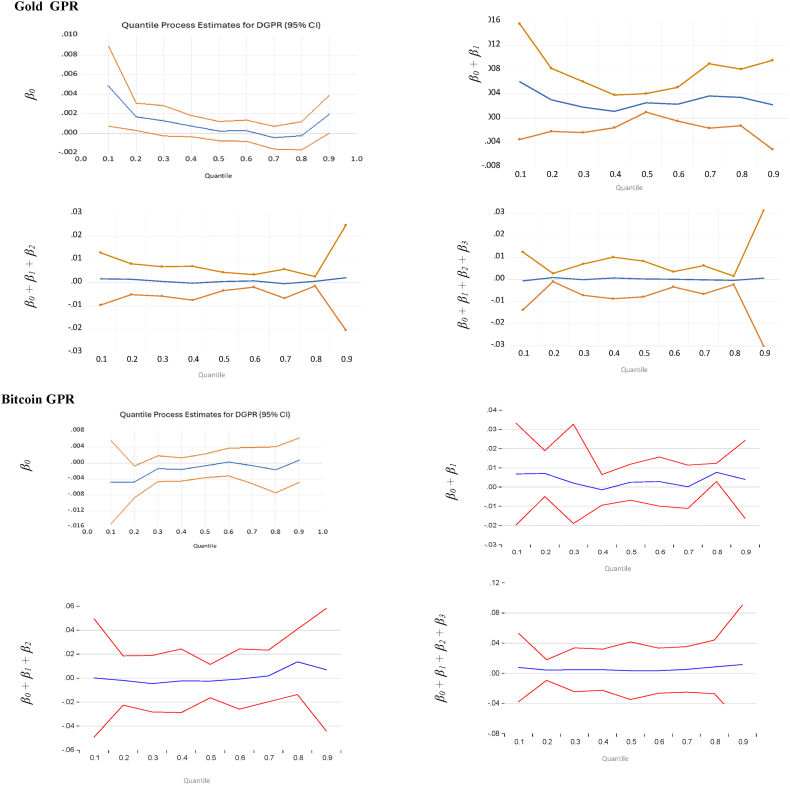

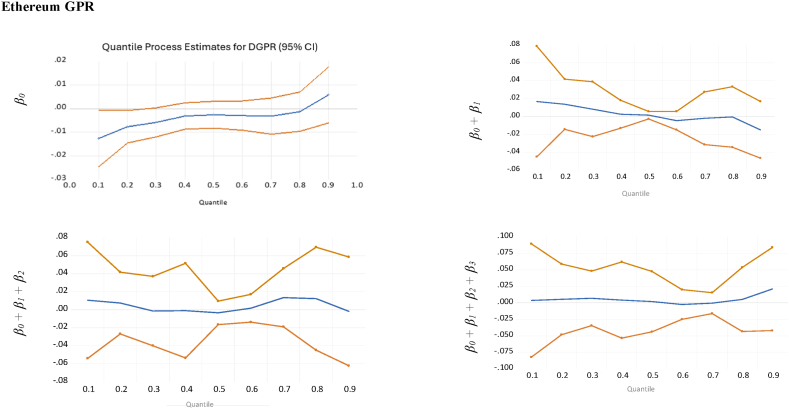
Quantile regression results with 95 % confidence bands for the effects of the change of GPR on Gold, Bitcoin, and Ethereum at extreme 90 %, 95 %, and 99 % quantiles of GPR.

We also evaluate the hedge and safe-haven abilities of gold and cryptocurrencies with 95 % confidence bands related to Eq. (5), present in Fig. 1, Fig. 2, respectively. It is noticeable that gold provides more stability and reacts strongly to uncertainties, notably at the upper quantiles. In extremely bearish and bullish markets, both gold and cryptocurrencies act as weak hedges against uncertainties. Gold consistently reveals strong safe-haven properties against IDEMV and GPR during the extreme bearish and bullish markets, while cryptocurrencies provide mixed results (weak and strong safe-haven).

## 5 Conclusion

Recently, the pandemic outbreak required verifying and re-investigating the safe-haven properties of gold and two major cryptocurrencies. Several extreme market turmoils, including the COVID-19 pandemic, created an unprecedented upsurge in the two most important markets, notably gold and cryptocurrencies. Our study aims to re-examine and compare the abilities of financial assets against IDEMV/GPR shocks during bearish and bullish markets. We investigate whether the potential safe-haven or hedge properties significantly change when considering smaller and larger IDEMV/GPR shocks.

Our results demonstrated that gold consistently exhibited the role of a strong safe-haven against both the lowest and the highest IDEMV during the extremely bearish and bullish market, respectively. In contrast, cryptocurrencies can act as a strong safe haven only against low IDEMV during extreme bearish markets. Gold and bitcoin can act as weak hedges against the lower IDEMV. Further, we found that gold establishes a strong hedge capacity against extreme geopolitical events, while cryptocurrencies provide a weak hedge. Accordingly, gold consistently reveals strong safe-haven properties against GPR in extremely bearish and bullish markets. Cryptocurrencies’ hedging and safe-haven abilities are diverse across markets (weak and strong safe-haven).

In fact, during several turbulent periods, using cryptocurrencies as a hedge or safe-haven instruments would not be recommended. Cryptocurrencies are more responsive to big shocks such as covid 19 [19,36,42,63]. During periods of turmoil, gold keeps stability with a safe-haven, and hedge coefficients can reduce portfolio risks. Then, we suggest that Portfolio managers should consider evolving asset allocation strategies that leverage gold's strong safe-haven properties during periods of high geopolitical risk and market stress. Effective risk management frameworks should incorporate gold's stable performance to mitigate portfolio risks during severe geopolitical and market crises. Policymakers ought to appreciate gold's significance for preserving economic stability during crises and advocate for policies that enhance economic resilience against geopolitical disruptions. Furthermore, investors should focus on gold in their portfolios rather than overweighing investments in cryptocurrency markets, which require further examination.

Our research has some limitations, mainly due to data constraints. Additionally, our findings are based on specific time periods and market conditions, which might not apply to different geopolitical contexts or economic cycles. Future research should explore the volatile nature of cryptocurrency prices, particularly during unprecedented crises, and examine other potential refuge assets in various market conditions to provide a more comprehensive understanding of safe-haven dynamics. Future studies should extend our research by delving into the characteristics of the current unprecedented crisis and the volatile nature of cryptocurrency prices.

## CRediT authorship contribution statement

**Hanen Ben Ameur:** Writing – review & editing, Writing – original draft, Visualization, Software, Resources, Methodology, Investigation, Funding acquisition, Formal analysis, Data curation, Conceptualization. **Fouad Jamaani:** Writing – review & editing, Writing – original draft, Validation, Supervision. **Mohammed N. Abu Alfoul:** Writing – review & editing, Data curation.

## Data and code availability statement

Data will be made available on request.

## Funding acknowledgment

This research was funded by 10.13039/501100006261Taif University, Saudi Arabia, Project No. (TU-DSPP-2024-234). The authors thank 10.13039/501100006261Taif University, Saudi Arabia, for supporting this work through project number (TU-DSPP-2024-234).

## Declaration of competing interest

The authors declare that they have no known competing financial interests or personal relationships that could have appeared to influence the work reported in this paper.

## Footnotes

1 Δ ln(X_t_) is the logarithmic difference or log return of the series at time t, ln(X_t_) is the natural logarithm of the value of the series at time t, l^n^(X_t−1_) is the natural logarithm of the value of the series at time t−1.
